# Role of Extracellular Vesicles in Cellular Cross Talk in Malaria

**DOI:** 10.3389/fimmu.2020.00022

**Published:** 2020-01-31

**Authors:** Kehinde Adebayo Babatunde, Bibin Yesodha Subramanian, Ambroise Dioum Ahouidi, Paola Martinez Murillo, Michael Walch, Pierre-Yves Mantel

**Affiliations:** ^1^Center for Engineering in Medicine, Harvard Medical School, Massachusetts General Hospital, Boston, MA, United States; ^2^Department of Oncology, Microbiology and Immunology, University of Fribourg, Fribourg, Switzerland; ^3^Laboratory of Bacteriology and Virology, Le Dantec Hospital, Cheikh Anta Diop University, Dakar, Senegal; ^4^Institute for Health Research, Epidemiological Surveillance and Training (IRESSEF), Dakar, Senegal; ^5^Department of Pathology and Immunology, University of Geneva, Geneva, Switzerland

**Keywords:** extracellular vesicles, Malaria, cellular communication, Plasmodium faciparum, Infection

## Abstract

Malaria infection caused by the Plasmodium species is a complex disease in which a fine balance between host and parasite factors determine the disease severity. While in some individuals, the infection will trigger only a mild and uncomplicated disease, other individuals will develop severe complications which lead to death. Extracellular vesicles (EVs) secreted by infected red blood cells (iRBCs), as well as other host cells, are important regulators of the balance that determines the disease outcome. In addition, EVs constitute a robust mode of cell-to-cell communication by transferring signaling cargoes between parasites, and between parasites and host, without requiring cellular contact. The transfer of membrane and cytosolic proteins, lipids, DNA, and RNA through EVs not only modulate the immune response, it also mediates cellular communication between parasites to synchronize the transmission stage. Here, we review the recent progress in understanding EV roles during malaria.

## The Plasmodium Life Cycle

Pathogens have developed successful strategies to survive in a hostile environment. Malaria is an infectious disease with an enormous public health impact worldwide (1). According to the WHO, almost half of the world's population is under threat of being infected with malaria. The malaria parasite, *Plasmodium*, has demonstrated a considerable capacity to persist and expand despite many years of control efforts. It is very concerning that 97 countries still have active malaria transmission in 2017 (2). There are five species of the protozoan genus *Plasmodium* known to infect humans: *P. falciparum, P. vivax, P. malariae, P. ovale*, and *P. knowlesi*. In total, the parasites caused over 219 million cases of malaria infection in 2017. The estimated number of malaria deaths stood at 453,000 in 2017, recorded majorly in children in Sub-Saharan Africa. While *P. falciparum* is the most deadly parasite, *P. vivax* is becoming increasinly recognized as a deadly pathogen as well (3).

**Figure 1:**
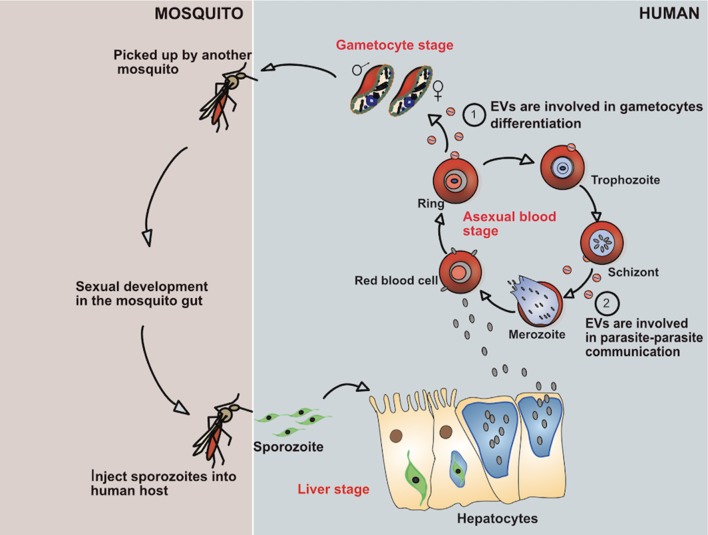
Showing life cycle of *P. falciparum* parasite and how it evades the immune system by secreting EVs.

The malaria parasites have a complex life cycle requiring a human and mosquito host (Figure 1). The transmission of malaria parasites is through an infective bite from a female Anopheles mosquito, by which sporozoites enter into their human hosts (4). After migration of the sporozoites from the infective bite, the pre-erythrocytic developmental stage is initiated in the liver where the released sporozoites infect the hepatocytes in a process known as the liver stage. Within the hepatocytes the parasites develop, protected by the parasitophorous vacuole, where they replicate as hepatic schizonts over a period of 10–12 days. Ultimately, upon release from the parasitophorous vacuole as tens of thousands of merozoites, the merozoites rapidly invades RBCs in the bloodstream to start the blood stage of the infection. Merozoites differentiate and replicate inside the parasitophorous vacuole to produce more daughter merozoites. At this stage, the replication of merozoites produces between 15 and 30 daughter parasites which are then released from the host cell upon parasite egress and subsequently re-invade RBCs to start a new asexual replication cycle (5). In humans, the asexual cycle can result in the infection of more than 10% of the total RBCs. During the blood stage, a small proportion of the parasites will eventually differentiate into gametocytes to begin the sexual cycle which are subsequently taken up by mosquitos during the next blood meal (6).

After the gametocytes are picked up by the female anopheles mosquitoes, the sexual development phase starts in the midgut, where the parasites produce gametes (7). During gametogenesis, the male microgametes and female macrogametes fuse to give rise to a zygote which transforms into a motile and invasive ookinete, which quickly exits the midgut lumen and traverses the mosquito mid-gut wall to finally become a sessile oocyst (8). Then, the parasite settles down at the basal site of the midgut epithelium and replicates asexually to produce thousands of infective sporozoites. Following egress, the sporozoites migrate from the ruptured oocyst and invade the mosquito salivary glands, to be released upon the next mosquito bite into the dermis of a human host. This completes the life-cycle of *Plasmodium* (4).

## What are the Molecular Mechanisms Leading to Severe Malaria?

Whereas, the liver stage remains clinically silent, the blood stage causes the symptoms and pathology observed during malaria infections. Parasites have developed several ways to evade the immune system and avoid destruction (9). The parasites are masters in immune regulation and immune avoidance (10). For instance, iRBCs, by adhering to dendritic cells, inhibit their maturation into fully functioning antigen-presenting cells and subsequently diminish their capacity to stimulate T cells. Infected RBCs express a family of variable antigens called *P. falciparum* eryhrocyte membrane protein 1 (PfEMP1), which are exported by the parasite to the surface of the iRBCs. PfEMP1 is a virulence factor that plays a key role in immune regulation and immune evasion. It is well-known that PfEMP1 is essential for the parasite survival *in vivo*. First, PfEMP1 is responsible for the inhibition of dendritic cell maturation by interacting with the CD36 receptor (11). In addition, PfEMP1 suppresses the production of the cytokines IFN-γ by γδ-T cells, αβ-T cells, and NK cells (12). Besides its role in inhibiting immune cells, PfEMP1 promotes the binding of iRBCs to the endothelium of capilaries, and therefore enables the parasites to avoid clearance of mature parasites by the spleen (13, 14). *P. falciparum* express about 60 different variations of the PfEMP1 receptors encoded by the var gene family, which provides an efficient strategy to escape the humoral immune response (15, 16). Once under pressure from a PfEMP1-antigen specific antibody response, the parasites can survive by switching to the expression of another PfEMP1 variant. Despite the arsenal of tools deployed to manipulate and suppress the immune system, the parasites release pro-inflammatory factors that trigger a pro-inflammatory response orchestrated in particular by the innate immune system. In fact, it is believed that parasite factors interacting with innate immune cells, including macrophages and dendritic cells, initiate the pro-inflammatory response. Several parasite factors have been identified to activate the innate immune system, such as glycosylphosphatidylinositol (GPI) and hemozoin. GPIs constitute a distinct class of glycolipids that are found ubiquitously in eukaryotes and are involved in many biological processes (17). GPIs anchor proteins that are essential and abundant in *Plasmodium* (18). *Plasmodium* GPI anchors trigger TLR2, which results in pro-inflammatory cytokine release (19, 20). However, GPIs alone are not a robust immune activator agent; in fact GPI anchors are coated with plasmodial DNA which adds magnitudes of immune stimulatory potency (21–23). In addition, during the intraerythrocytic stage, parasites digest hemoglobin and produce hemozoin, an inert crystal. Since hemozoin is insoluble and toxic, it is then transported in food vacuoles in the iRBCs (24). Natural hemozoin is coated with both plasmodial DNA and proteins. Once hemozoin is detected by the immune system, it stimulates TLR9 (25–27). The immunostimulatory properties of hemozoin to activate TLR9 are probably due in part to the delivery of plasmodial gDNA to the endosomal compartment of the immune cells. The gDNA is essential for immune activation, since upon DNAse treatment of hemozoin the activation is lost (25–27).

The genome of *P. falciparum* with 80% AT-rich is remarkably unusual and is recognized by the human immune system (28). Indeed, the AT-rich stem-loop DNA motif derived from the malaria genomic DNA sequences stimulates the release of Type I IFNs by immune cells by signaling through TLR9 (26, 29). As demonstrated by using immune cells from different mouse knock-outs, the cytokine release requires the stimulator of interferon genes (STING), Tank-binding kinase 1 (TBK1), and the interferon regulatory factors 3 (IRF3) and IRF7 (30–32). While it is believed that hemozoin and GPI anchors are released into the bloodstream during parasite egress (33), the mechanism involved in *P. falciparum* gDNA delivery from iRBCs to the cytosol of host immune cells is not clear. Recently, it was shown that *P. falciparum* extracellular vesicles (EVs) contain plasmodial gDNA that can trigger host cytosolic innate immune cell receptors upon uptake of EVs (34). EVs (which are also known as exosomes, microparticles, and microvesicles) are fragments shed almost spontaneously from the plasma membrane blebs of virtually all cell types. The release of EVs increases when the cells are submitted to a number of stress conditions (35). In addition to GPI anchors and hemozoin, small vesicles secreted not only by iRBC, but by any type of cells, are suggested to participate in the pathogenesis of malaria (36).

### Types and Origin of Extracellular Vesicles

EVs are a heterogeneous group of cell-derived membranous structures that are divided into two subgroups based on their size and biogenesis, namely exosomes and microvesicles. Exosomes are vesicles that range from 30 to 150 nm in diameter, which are released by reticulocytes during, for example, differentiation. (37). They are originally formed as intraluminal vesicles (ILVs) within endosomal multivesicular bodies (MVBs) and secreted upon fusion of the MVBs with the plasma membrane (38). Microvesicles range in size from 150 nm to 1–2 μm in diameter, and are generated by the outward budding and fission of the plasma membrane into the extracellular spaces (39). The content of EVs include lipids (40, 41), proteins (42, 43), nucleic acids, including RNAs [mRNAs and noncoding RNAs, including microRNAs (miRNAs, tRNA, Y-RNA)] (44, 45), and DNA sequences (46).

### Cellular Origins of EVs During Malaria

*P. falciparum* infection is the main source of life-threatening malaria. Cerebral malaria (CM) developed in about 1% of *P. falciparum* infections, and is the leading cause of death (47, 48). Early CM diagnosis remains challenging, and despite available treatment, 15–20% of patients die, while 10–15% of cured patients will suffer from long-term neurological deficits (49). Plasmodium iRBCs sequester in capillary beds of deep tissues, through adhesion to vascular endothelium (50). While cytoadhesion provides a way for the mature parasites to avoid clearance by the spleen, it promotes local inflammation. During inflammation, EVs are released from almost all cell types. Activation of microvascular endothelium in a variety of disease states (such as severe sepsis) by the cytokine TNF-alpha causes the release of endothelial EVs into circulation, (51). Therefore, EVs are also associated to a variety of pathophysiological conditions in which TNF-alpha is involved (52). The plasma concentrations of TNF-alpha are increased in patients with malaria, which results in a significant elevation of circulating endothelial EVs in malaria patients with different degrees of disease severity. The number of circulating endothelial EVs were significantly elevated in children suffering a coma and severe malaria in a study performed in Malawi. Interestingly, during convalescence, the number of endothelial EVs diminished compared to the acute stage in the malaria patients (53).

Endothelial EVs released from endothelial cells in cell culture have both prothrombotic and proinflammatory capacities (51). Therefore, the abundant circulating endothelial EVs in patients with severe malaria may contribute to the pathogenesis by promoting the widespread deposition of fibrin and activation of platelets observed during the deadly CM (54).

Insights into the functional role of endothelial EVs in the pathogenesis of malaria *in vivo* come from the rodent malaria model. EVs were generated *in vitro* by treatment of mouse endothelial cells with TNF-alpha stimulation to obtain a very pure population of EVs. Then, the purified endothelial EVs from resting or TNF-stimulated endothelial cells were injected into healthy mice to determine their contribution to the CM lesions. Interestingly, the injection of endothelial EVs from TNF-stimulated cells led to worsening pathology, and induced CM-like brain injuries (55). Thus, endothelial EVs are likely to contribute to the local inflammatory process as well as the pathology.

Although an increased release of endothelial EVs correlates with malaria severity, other cell types release EVs, including platelets, monocytes, T lymphocytes, and, most importantly, RBCs. Several studies have included additional markers to identify the cellular sources of EVs. In a study performed with patients infected with *P. falciparum* recruited in Cameroon, the platelet, RBC, endothelial, and leukocyte EVs were elevated in patients with cerebral dysfunctions and returned to normal by discharge. EVs quantification and phenotyping were carried out by flow cytometry using the following cell-specific markers to identify the cellular sources of EVs: CD41, CD105, CD235a, CD51, CD11b, and CD3 to quantify EVs derived from platelets, RBCs, endothelial cells, monocytes, and lymphocytes, respectively (56). In CM patients, platelet EVs were the most abundant and their levels significantly correlated with coma depth and thrombocytopenia. Platelet EVs were also the most elevated in a cohort of 37 uncomplicated *P. vivax* infections from the Brazilian Amazon. RBC and leukocyte EVs were increased as well when compared to healthy donors. The markers used to measure EVs originating from platelets, RBCs, leucocytes, endothelial cells, and monocytes were CD41a, CD235a, CD45, CD144, and CD14, respectively (57). Importantly, the level of EVs decreased after 21 days with anti-malaria treatment.

Interestingly, platelet derived EVs are known to have pro-inflammatory properties. For example rheumatoid arthritis is a chronic inflammatory disease that causes swollen joints and organs, and may therefore contribute to malaria pathogenesis (58). Joint fluid of patients with rheumatoid arthritis contained platelet EVs that trigger Interleukin-1 secretion from synovial fibroblasts. In fact, platelet EVs may be the inflammatory trigger in the conflagration of a swollen and painful rheumatoid joint (59). Thus, similarly as for rheumatoid arthritis, platelet EVs might contribute to the pathology of *P. vivax* infections. These data are in accordance with the observation that active, non-cytokine factors are circulating in plasma with acute *P. vivax* infections during paroxysm (60, 61).

In a study performed in Thailand, the highest levels of RBC EVs were found in patients with severe *P. falciparum*, whereas *P. vivax* and *P. malariae* were less increased. Interestingly, the parasite protein Ring-infected erythrocyte surface antigen (RESA) was detected on EVs by Flow Cytometry and demonstrated that the iRBCs produce about 10 times more EVs than RBCs (56, 62). A study in India found the EV level to be correlated with TNF-alpha levels and to be significantly elevated in febrile malaria patients (63). In all those studies, the EVs were quantified from plasma by using flow cytometry (64, 65). The small size and heterogeneity of EVs make the vesicles particularly difficult to detect by flow cytometry, since EVs are at the limit or even below the limit of detection. It is possible that the smaller EVs were not detected in those studies. However, the development of new technologies, such as the nanoflow that allow detection of exosomes, will improve the detection and the characterization of the EVs throughout this complex disease (66).

Altogether, the strong correlation of EVs with disease severity suggests a role of EVs in the development of the malaria pathogenesis. Importantly, EV levels go back to normal after drug treatment and clearance of the parasites, suggesting that EVs might be used as biomarkers to predict the severity of the disease (67).

## Extracellular Vesicles in the Pathogenesis of Severe Malaria

What are the parasite-derived elements that trigger the pro-inflammatory innate immune responses during malaria infection? Recent work in the rodent malaria model suggests that EVs are potentially the source of circulating parasite factors. First, animals deficient in EV production either genetically or pharmacologically do not develop cerebral malaria (68, 69). Second, EVs released during infections generate a strong pro-inflammatory response (70). Third, adoptive transfer of EVs isolated from plasma of *P. berghei ANK*A-infected mice caused the destruction of the blood brain barrier integrity (55).

The blockade of EV production genetically significantly diminishes the inflammation and the resulting disease severity. The transbilayer transport of phosphatidylserine (PS) to the external leaflet of the cellular membrane is mediated by the ATP-binding cassette transporter A1 (ABCA1). The external exposure of PS seems to be a mandatory step for EV shedding. In humans, the loss of a functional ABC1 protein leads to an autosomal recessive disorder of lipid metabolism known as Tangier disease (71–74). Exposure of PS upon Ca^2+^ stimulation is impaired in ABCA1-/- mice. In fact, the ABCA1 knockout mice exhibit lower amounts of PS after incubation with A23187, when compared to WT mice, as measured by prothrombinase activity and EV production (75). Moreover, ABCA1 overexpression in cell lines result in increased levels of cholesterol-rich EV release (76). When mice were infected with *P. berghei ANKA*, the ABCA1 -/- mice did not develop neurological syndromes and were fully protected against CM. The ABCA1-/- suffered from reduced inflammation as compared with the WT mice, which was accompanied with a significantly lower level of plasma TNF-alpha. Finally, histological analysis revealed a reduced number of perivascular hemorrhages with no evidence of vascular sequestration of immune cells (68). Interstingly, a study found that polymorphisms in the human ABCA1 promoter correlate with the severity of the disease (77), further suggesting a role for ABCA1 in controlling the level of pro-inflammatory EVs during malaria disease.

In addition, cerebral syndrome in *P. berghei* ANKA-infected mice is prevented by pharmacological blockade of EV production by the administration of the dietary provitamine pantethine, a key regulator of lipid metabolism (78). The pantethine conferred protection is accompanied with an unadapted host response to the parasite infection, with a reduction of circulating EVs and preservation of the blood-brain barrier integrity in particular. Importantly, the parasite growth was not affected by pantethine (69). Pantethine and ABCA1 act through different pathways, since ABCA1 activity was unaffected by the treatment with pantethine (69). Altogether, these results suggest a pro-inflammatory effect and pathogenic role of EVs, contributing to the development of the neurological syndromes.

The release of a strong pro-inflammatory type-1 immune response is correlated with the clinical manifestations of severe malaria (79). Although early innate and adaptive inflammatory responses provide an essential protection mechanism for fighting against malaria parasites, excessive and uncontrolled secretion of pro-inflammatory cytokines, including IL-6, TNF, and IFN-gamma, may also directly result in severe pathology, including severe anemia, CM, and organ damage. All cytokines that are induced by immune cells when treated with EVs are pro-inflammatory, further suggesting a role of EVs in triggering an excessive and uncontrolled pro-inflammatory response. Indeed, incubation of macrophages *in vitro* with EVs derived from the plasma of *P. berghei* infected mice resulted in TNF-alpha secretion and the expression of CD40 on the cell surface. Interestingly, these EVs were a more potent stimulant of macrophage activation than intact iRBCs *in vitro* (70). Similarly, EVs derived from *in vitro* cultures of *P. falciparum* iRBCs activated the secretion of IL-6 and TNF-alpha by human macrophages (80).

While inhibition of EV production genetically or pharmacologically protects against the development of CM *in vivo*, adoptive transfer of EVs into mice exacerbated the neurological symptoms (55). To track EV faith *in vivo*, EVs isolated from mice infected with *P. berghei ANKA* were fluorescently labeled and subsequently injected into mice. EVs disappeared very rapidly from the blood circulation and were cleared within minutes of injection. However, microscopic tissue examination revealed arrested EVs along the endothelium within the lumen of brain vessels of infected animals, whereas they were absent from healthy recipient mice. This suggests a role for EVs in priming the immune system toward a pro-inflammatory response.

Altogether, these data provide strong evidence that EVs contribute to the onset and exacerbation of severe malaria by triggering a strong pro-inflammatory response. However, the exact nature and composition of the EVs during infection remains elusive.

### Different Types of Vesicles

EVs are composed of a heterogeneous group of cell-derived vesicles including exosomes and microvesicles (MVs). EV classification is mainly based on differences in biogenesis and the mechanisms of release of the vesicles. Since in current experimental settings it remains almost impossible to distinguish between the different types of EVs, they are thus commonly called EVs (35, 81). Exosomes are small vesicles of a size comprised between 20 and 100 nm. They originate from the endosomes and are released following the fusion of the multivesicular bodies (MVBs) with the cellular membrane (82). The term exosome was used first to refer to membrane vesicles released by reticulocytes during RBC maturation (37, 83, 84). However, most cell types are likely to release both exosomes and MVs, particularly under the right stimuli. Therefore, in many studies EVs were named exosomes and are probably an heterogenous mixture composed of a population of exosomes and MVs (83, 84).

Whereas, exosomes are small, the terms MVs, microparticle, and ectosome refer to larger, 100-1,000 nm vesicles originating from the plasma membrane (85, 86). MVs were initially known as “platelet dust” and were first described as subcellular material originating from platelets in normal plasma and serum (87). MVs play a role in intercellular communication in various cell types and are shed by the outward budding and fission of the plasma membrane and the subsequent release of vesicles into the extracellular space.

Most biological fluids contain EVs, including plasma, urine, saliva, semen, and breast milk (88). The role of MVs is now evident in an increasing number of physiological and pathological processes (89).

In a complex disease such as malaria, it remains difficult to fully determine the composition, cellular origin, and contribution of the EVs to the parasite biology and to the development of the pathology. However, the plasma of malaria-infected individuals is likely composed of an heterogenous mix of exosomes and MVs. Furthermore, EVs are originating from different cellular origins, so their composition and function might change with the evolution of the infection. EVs isolated from *in vitro* culture of *P. falciparum* iRBCs were composed of human RBC proteins as well as parasite proteins (80).

Antwi-Baffour et al., isolated EVs by centrifugation from plasma of forty-three patients infected with *P. falciparum* and carried out proteomics; they found a significantly larger amount of hemoglobin in the EV fraction from infected patients when compared to uninfected, suggesting that RBCs infected with *P. falciparum* lose their hemoglobin to vesiculation. Interestingly, several parasite proteins were identified including Pfenolase, Pfactin, as well as heat shock 70 kDa protein (Hsp70). The following plasmodial proteins were found only in a few samples: RESA, Apical membrane antigen 1 (PfAMA1), and Merozoite surface antigen 1 (MSA-1) (90). Furthermore, Abdi et al., using patients from Kenya, defined the core parasite secretome of *P. falciparum*-derived EVs with clinical isolates via proteomic analysis. They showed that *P. falciparum* EVs are enriched in proteins found both within the exomembrane compartments and secretory endomembrane compartments of iRBCs and the apical end of the merozoites respectively, thereby suggesting that these proteins play a role in parasite-host interactions (91).

Parasite proteins were also identified on EVs in the mouse malaria. The rodent CM model was used to analyze the protein composition by proteomics to compare EV proteins from non-infected and *P. berghei ANKA*-infected mice. Of the more than 360 proteins identified, 60 were differentially abundant when comparing between non-infected vs. *P. berghei ANKA*-infected mice. Furthermore, network analyses showed that CM EVs carry proteins involved in molecular mechanisms relevant to CM pathogenesis, including endothelial cell activation. Although only two parasite proteins were identified= (intra-erythrocytic *P. berghei*-induced structures protein 1 and merozoite surface protein-1), the two host proteins, carbonic anhydrease 1 and S100A8, were strictly associated with the disease severity (92). A previous study on EVs secreted during *P. yoelii* infections identified a larger number of parasite proteins in the EVs (93).

## Cell-Cell Communication During Malaria

Living organisms have developed cell-to-cell communication to maximize their survival and development potential. Several modes of communication are known, including release of soluble signaling factors, cell-to-cell contact, and, finally, signaling cargoes transferred via EVs. EVs play several roles in normal physiological processes, as well as in diseases such as cancer and infectious diseases (94). EVs were long considered as cellular garbage, only necessary for the cells to get rid of unnecessary molecules and organelles. However, the discovery that EVs contain functional cargoes that can be transferred from a donor to an acceptor cells boosted the field of cellular communication. In fact, EVs contain both functional miRNA and mRNA, and these EV-incorporated mRNAs can be translated into proteins by target cells, while miRNAs regulate gene expression in the recipient cell (95, 96).

EVs released by immune cells selectively incorporate functional miRNA that can be transferred after fusion with the recipient cells (97, 98). In addition, EVs contain a variety of other small noncoding regulatory RNA species, including structural RNAs, tRNA fragments, vault RNA, Y RNA, and small interfering RNA (44, 45, 99). It is worth noting that RNA isolates from EVs are relatively enriched with different RNA profiles than the parent cells (44, 95, 96, 100), suggesting that cellular mechanisms exist to selectively incorporate RNA molecules into EVs (101). In addition to RNAs, EVs can transfer other functional cargoes, including proteins, DNA, and lipids to a recipient cell; all of these molecules can be involved in immune regulation or cellular communication.

Both pathogen-derived EVs and host-cell derived EVs contribute to the pathogenesis of parasitic diseases (102). In order to adapt and survive in their host cells, bacteria, fungi, and parasites have developed strategies to manipulate their surroundings. Pathogens release EVs to modulate the immune system of their host to promote their survival. For example, *Leishmania donovani* transfers GP63 in EVs to target pre-miRNA processor Dicer1 to downregulate miR-22 and lower serum cholesterol, thereby promoting the parasite survival (103). Epstein-Barr viruses infected B cells shuttles functional mature EBV-encoded miRNAs in EVs to immature monocyte-derived dendritic cells to downregulate the expression of CXCL11/ITAC in primary EBV-associated lymphomas (104). The malaria EVs contain functional human miRNAs (45, 105), of which the most abundant is miR451a. This miRNA is essential for the final steps of RBC maturation (106, 107). The EVs contain the mature form of miR451a, which forms a RNA-induced silencing complex (RISC) with Argonaute-2 (108, 109). Remarkably the miR451a complex was able to cleave specifically an RNA probe in an *in vitro* RNA cleavage assay. EVs were rapidly picked up by endothelial cells in culture *in vitro* and had a strong effect on endothelial cell function resulting in an increase in permeability (110). This might be relevant during CM and the accompanied blood brain barrier degradation. The EV transferred miR451a was targeting the expression of genes involved in vascular permeability. Therefore, the increased EV level observed in the plasma of patients with severe malaria might affect blood brain barrier integrity by delivering miR451a to the endothelium (111–113). The resulting vascular dysfunction may lead to endothelial cell activation and further sequestration of iRBCs to the endothelium into the microvasculature of the brain. Cellular aggregates of uninfected and iRBCs cause clogging of blood microvessels and damage blood vessel walls, leading to microhemorrhages associated with high levels of circulating pro-inflammatory chemokines and cytokines. The microbleedings provide contact between parasite products and brain resident cells. While during CM, the astrocytes and microglia are activated and contribute to the production and release of several mediators, during neuroinflammatory processes the processes involved in their activation are not known. EVs are rapidly transferred from the rodent parasites to astrocytes, and to lesser extent to microglia. Activation with EVs leads to an increase of interferon gamma inducible protein 10 (IP10) (114). IP10 is a pro-inflammatory cytokine, known to promote the recruitment of pathogenic CD4 and CD8 T lymphocytes within the brain, and participate in the pathophysiology of the rodent malaria (115, 116).

Malaria EVs contain small non-coding regulatory RNAs (45, 80, 105), as well as plasmodial gDNA (34). EVs transfer plasmodial gDNA from the parasites to human monocytes, where it stimulates STING. STING is an immune adaptor in the cytosol of innate immune cells that detects microbial DNA. Following internalization of EVs, *P. falciparum* gDNA is released inside the host cell cytosol, resulting in STING-dependent DNA detection and promoting a pro-inflammatory response by secretion of cytokines (34).

### Immune Cells and EVs

Immune cells like Natural Killer (NK) cells are important in mounting immune responses against malaria parasite infection, but seem to elicit significant differences in their responses in the human population. In a study by Ye et al., they demonstrated that NK cells that elicit immune response to iRBC expressed increased pathogen recognition receptor, MDA5. These receptors on NK cells are activated by iRBC-derived extracellular vesicles. This implies that non-responding NK cells during malaria infection can be improved by activating MDA5 receptors with EVs, which thereby could potentially serve as a NK cell-based intervention of malaria infection in humans (117).

Proteomics analysis of EVs revealed the presence of several vaccine candidate proteins associated with EVs. Interestingly, immunization of mice with EVs protects them upon infection with *P. yoelii* against the development of severe disease. Although the immunization did not provide full protection against the parasites, it protected against the cerebral symptoms (93).

While most investigations demonstrated a proinflammatory role of EVs, some other works instead describe an immune suppressive effect. In fact, EVs might have different composition and therefore different properties depending on the timing of release during the maturation of the parasite. For example, the parasite virulence factor PfEMP1 is detected in EVs only up to 12 h post-RBC invasion (118). When human primary monocytes are treated with EVs from PfEMP1 deficient parasites, they express more genes associated with the defense mechanisms, which suggests a role for EV PfEMP1 in suppressing the immune response (118).

Furthermore, in the rodent malaria model, EVs inhibited the proliferative response of CD4+ T cells in response to antigen presentation. Two proteins associated with EVs, the histamine releasing factor and the elongation factor 1 alpha, have immunosuppressive activities. Interestingly, when these two proteins are genetically deleted from the parasites, EVs fail to suppress T cell responses *in vitro* and *in vivo* (119).

The immune modulatory nature of malaria EVs seem complex, and the overall effect on the immune cells might very well depend on the concentration, composition of EVs, and host factors such as cytokines and chemokines that are produced during the infection.

## Parasite-Parasite Communication: a Sexual Commitment

Cell-to-cell communication plays a key role in maintaining the homeostasis of complex multicellular organisms. Similarly, unicellular organisms, including bacteria and yeasts, secrete small molecules used to communicate with each other at the population level. These signaling molecules work as sensors to assess nutrient conditions and environmental stresses. Cellular communication allows the microbes to adapt their growth in order to optimize population survival (120).

During the blood stage of an infection with *P. falciparum*, a small number of parasites differentiate into gametocytes that are necessary for transmission to the mosquito vector. The stress conditions caused by high density of parasites, exposure to drugs, or the host immune response can all contribute to the induction of this once in a life-time commitment. Although differentiation into gametocyte can be induced by factors released by parasites, these remain unknown (121). In fact, parasite culture supernatants can lead to greatly increased numbers of gametocytes *in vitro*. EVs released from iRBCs are important components of this conditioned medium. Once depleted of EVs, the conditioned medium is less efficient in inducing gametocytes. Remarkably, EVs are internalized by iRBCs and stimulate conversion to the sexual parasite cycle in a titrable fashion (80, 122). Therefore, the malaria parasites have developed means to sense the EV concentration in its surroundings to optimize its increasing commitment to gametocytes. Therefore, in analogy to the well-described quorum-sensing used by bacteria, the malaria parasites promote their differentiation into the sexual forms and escape to the mosquito vector in response to conditions under stress in the host. For example, increased biogenesis and release of EVs under drug-related pressure could serve as a means of surviving and reacting to stress conditions. Therefore, the accumulation of EVs in the plasma of infected individuals may constitute a signal that initiates and promotes the development of the transmission stage parasites to maximize their transfer to the mosquito vector.

Although the exact EV cargoes and mechanisms of gametocytogenesis initiation are yet to be identified, we know that EVs could be a key player in communication between parasites, since they are known to shuttle signaling cargoes including messenger RNA, microRNA, small non-coding RNA, lipid mediators, and proteins (34, 45, 80, 105, 123, 124).

So far, EVs have been demonstrated to serve as a shuttle for plasmids carrying genes encoding drug resistance markers from one *P. falciparum* iRBC to another without requiring cell contact (122). Although EVs can transfer nucleic acids between parasites, the exact signaling cargoes responsible for the gametocyte commitment have not been identified yet. Nonetheless, the Maurer's Clefts, organelles found in iRBCs, seem to be necessary for parasite—parasite communication. In a genetic screen, Maier et al., identified several parasite proteins that are essential in the secretion of *P. falciparum* variant surface antigen (PfEMP1) to the cell membrane of iRBCs. Deletion of one of the genes that encodes for PfEMP1 trafficking protein 2 (PfPTP2) in parasites lead into the full blockage of both the release and uptake of parasite-derived EVs. PfPTP2 is localized to vesicles budding from the Maurer's cleft in iRBCs (125).

Therefore, EV secretion allows parasites to communicate and sense when it is time for differentiation into gametocytes to prioritize transmission to the mosquito.

### Potential of EVs in Malaria Vaccine Development

The development of a potent vaccine against malaria still remains elusive because of the complexity of the *Plasmodium falciparum* parasite life cycle and large surface protein redundancy. The central role played by EVs in malaria could be potentially harnessed as a delivery mechanism for a malaria vaccine. Synthetic EVs encapsulated in nanovesicles could provide an effective therapeutic approach to anti-malarial vaccine development (126, 127). Furthermore, *in vivo* studies in mice have demonstrated the immunization potential of poly-lactic-*co*-glycolic acid (PLGA) coated iRBC-derived EVs (93). The delivery of *P. vivax* antigens using PLGA as a vehicle demonstrated promising and improved immune response when compared to standard vaccination technique (128). In addition, PLGA microparticles have been used to deliver *Plasmodium* antigen-encoding plasmid DNA to antigen-presenting cells (129). Targeting the sexual stage of the parasite by transmission blocking vaccines have been shown to be promising but less efficient. However, controlled slow release of loaded antigen in biodegradable micro-particles have been demonstrated to have sustained functional antibody responses and potentially make anti-malaria vaccine more potent (130).

## Extracellular Vesicles are Key in Other Parasitic Diseases

Much remains to be understood in the role of EVs during malaria infections. Besides the Plasmodium species, a large number of extracellular and intracellular parasites are releasing EVs as a mechanism of intercellular communication within the organism. Moreover, EVs are exported outside the organism resulting in host manipulation with profound consequences for parasite development, disease progression, and pathology in the host. Here, we discuss briefly the role of EVs during other parasitic infections in order to give new insights into the potential role of EVs in malaria research.

### Toxoplasma gondii

*Toxoplasma gondii* is an obligate intracellular opportunistic parasite that infects immunosuppressed patients and fetuses (131). *T. gondii* secretes EVs of sizes between 50 and 250 nm, containing a broad spectrum of proteins ranging from 15 to 70 kDa which have immunogenic responses. EVs from *T. gondii* also carry nucleic acids including mRNAs and small RNAs, such as miRNA. The miRNAs inside EVs act as potential immune regulatory mediators involved in the host cell manipulation (132). Treatment of macrophages with *T. gondii* EVs leads to the upregulation on one hand of the anti-inflammatory IL-10 and, on the other hand, the pro-inflammaotry TNF-α, IFN- γ, and iNOS. The induction of cytokine is dependent on the activation of the JNK pathway, thus eliciting an innate immune response which could be an essential regulator of host-pathogen interactions during *T. gondii* infection (133). In a study using the mouse model of *T. gondii*, when mice were immunized with host dendritic cell-derived EVs that were pre-treated with parasite cells or parasite antigens elicited a protective immune response to subsequent parasite challenge. This novel vaccination strategy against toxoplasmosis was efficient and triggered both a systemic and a local humoral response against the parasite *in vivo*.

### *Leishmania* spp.

Leishmaniasis in human and other animals is caused by the intracellular protozoan parasite which belongs to the Leishmania species. Leishmania species target a large range of mammalian hosts and settle within the host immune cells to avoid damage control by the host immune responses. Leishmania can release EVs through budding from both the flagellar membranes and the cell body. Proteomics analysis of EVs from *Leishmania donovani, L. mexicana*, and *L. major* secreted proteins has revealed several proteins that are linked to host immune modulation and parasite virulence factors. A change in temperature to simulate the host environment of *L. mexicana* is enough to induce a rapid protein secretion and release EVs from the *Leishmania* parasite in culture. These released EVs carry proteins that block essential macrophage immunomodulatory functions as the parasite transforms important host cell signaling pathways. *Leishmania* GP63 is one such major exosome-enriched protein that impairs immune cell functions since *L. major* deficient in gp63 lose their immunomodulatory capacities of leishmanial EVs in comparison to *L. major* wild type (134). EVs released by *L. amazonensis* promastigotes activate the expression of IL-10 and IL-6 by rodent naïve macrophages during cutaneous leishmaniasis. The presence of LPG Lipophosphoglycan and GP63 in *L. amazonensis* EVs stimulates macrophages and B1-cells to produce cytokines. B-1 cells are innate-like B cells and, when incubated with EVs, harbor a diminished release of IL-10 as compared to naïve macrophages. Thus, EVs modulate the cytokine expression to control the immune response in favor of the parasite expansion and disease evolution (135).

### *Trypanosoma* spp.

The obligate *Trypanomsoma cruzi* is an intracellular parasite that causes Chagas disease. In many South and Central American countries, *T. cruzi* remains a major socio-economic obstacle to further development. *T. cruzi*'s life cycle is composed of mammalian hosts and insect vectors. EVs are released at different stages of *T. cruzi*'s life cycle, including epimastigote, metacyclic, and tissue-derived trypomastigote stages. These EVs have a potent immunomodulatory capacity, and by interacting with immune cells, they promote immune evasion and parasite survival. In a mouse model of *T. cruzi*, injection of mice with EVs prior to infection by *T. cruzi* trypomastigote leads to a suppression of the immune response both locally and systemically. The immune dysfunction resulted in a transient and increased blood parasitic load. Normally, *T. cruzi* infection generates the production of nitric oxide (NO). However, when mice were injected with EVs prior to the *T. cruzi* challenge, they were not able to increase NO plasmatic levels. Thus, EV's stimulation had no direct activation capacity on host macrophages. However, it suppressed macrophage response, thereby setting up an environment more favorable for the pathogen with reduced NO production. EVs act indirectly by inducing the production of prostaglandin E2 (PGE2) by macrophages, which, at high concentrations, inhibits the release of pro-inflammatory cytokines (TNF-α, IFN-γ, IL-10, and IL-6) and reduced antigen presentation. Taken together, these data show that EVs from *T. cruzi* exerts a potent immunomodulatory effects on host immune cells during infection, promoting an environment more favorable to *T. cruzi* during the first steps of infection (136). In order to produce EVs, *T. brucei rhodesiense* build flagellar membrane nanotubes that dissociate and form vesicles leading to EV release. Similar to observations from *Leishmania* spp, EVs contain parasite virulence factors such as serum resistance-associated protein (SRA), Hsp-70, glycerol kinase, variant surface glycoprotein (VSG 221), and aldolase that contribute to the virulence of *T. brucei* in the mammalian host. Humans and other higher primates secrete the circulating trypanosome lytic factors (TLF) that protect them against many trypanosome species. The *T. brucei* are susceptible to killing by TLF. However, a *T. brucei* parasite expressing a Ty-epitope tagged with SRA are not only protected from TLF mediated killing, but they also produce EVs that carries the SRA protein on the membrane surface. Remarkably, upon incubation with SRA-Ty containing EVs, the wild type *T. brucei* not only became SRA-Ty positive, but also became resistant to TLF killing. This suggests that SRA is required for human infectivity, and *T. brucei rhodesiense* EVs transfer SRA to non-human infectious trypanosomes promote their immune evasion from the human innate immune responses. *T. brucei* EVs labeled with a fluorogenic dye were able to fuse with mammalian RBCs, suggesting that EVs transfer may also incorporate parasite virulence proteins to erythrocytes and make them targets of the immune system. Intravenous injection of EVs to mice led to an increased RBC volume accompanied with a 10.6% reduction in hematocrit causing severe anemia in trypanosome infected mice (137).

## Conclusion

**Figure 2:**
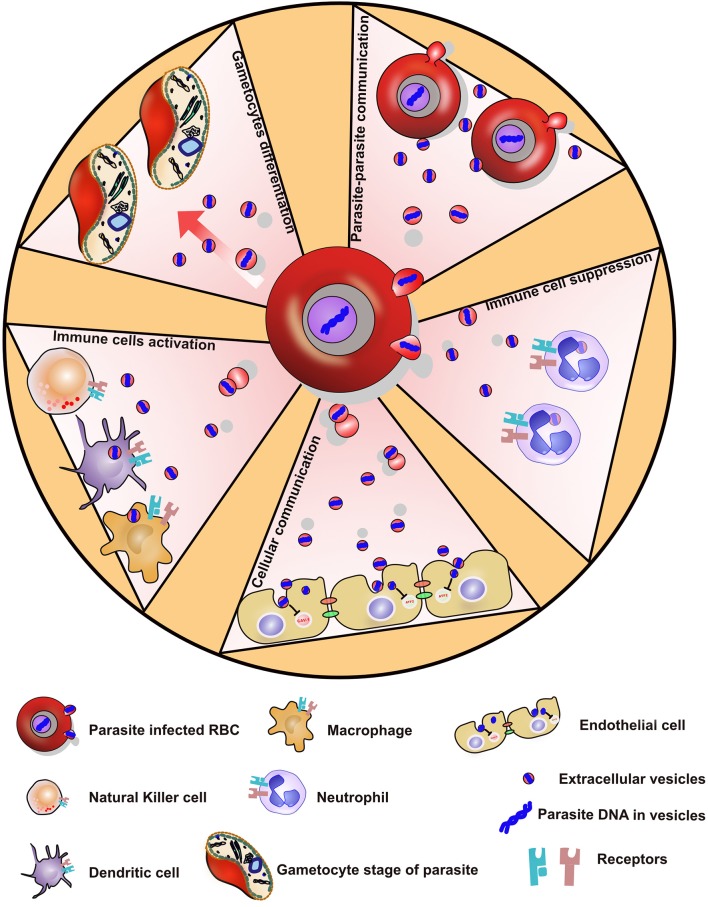
Showing various ways by which *P. falciparum* parasite evades the immune system via secreted extracellular vesicles.

Malaria infection is a complex disease that can cause moderate symptoms. However, some patients will develop severe syndromes resulting in death. Recently, several studies have investigated the numerous roles of EVs during malaria. It was demonstrated that EVs from various cell types including endothelial cells, RBCs, and platelets are elevated during a severe disease infection. Therefore, EVs might contribute to inflammation and to the pathogenesis. The malaria parasite during evolution has optimized its mechanisms of infection in order to survive in hostile environments. The small vesicles secreted by iRBCs are essential for the parasites to communicate and coordinate themselves to differentiate into gametocytes to be picked up by mosquitoes. By secreting EVs, the parasites might orchestrate at the population level, the transmission to the mosquito occurring when stress conditions are increasing in the host, and become more hostile to the parasites (Figures 1, 2). EVs contribute to the immune regulation by transferring cargoes including RNAs from the iRBCs to immune cells, resulting in immune suppression or immune activation depending on the cellular context. Furthermore, synthetic or biological EVs might potentially serve as a vaccine delivery mechanism to cure malaria, however further investigation is still needed to be done on the specific roles of these EVs during active malaria infection in humans. Taken together, these works point out the central role of EVs in malaria and highlight how a combination anti-malaria vaccine containing candidate antigens, delivered using EVs or nanovesicles, has the potential to be a promising and effective vaccination against malaria.

## Author Contributions

P-YM, KB, and BY drafted the manuscript. KB prepared the figures. AA, PM, and MW provided critical feedbacks.

### Conflict of Interest

The authors declare that the research was conducted in the absence of any commercial or financial relationships that could be construed as a potential conflict of interest.
